# Molecular characterization of genes responsible for biofilm formation in *Staphylococcus aureus* isolated from mastitic cows

**DOI:** 10.14202/vetworld.2022.205-212

**Published:** 2022-01-30

**Authors:** Eman Shafeek Ibrahim, Amany Ahmed Arafa, Sohad Mohamed Dorgam, Rasha Hamdy Eid, Nagwa Sayed Atta, Wahid Hussein El-Dabae, Eslam Gaber Sadek

**Affiliations:** 1 Department of Microbiology and Immunology, National Research Centre, Dokki, Giza, Egypt; 2 Udder Health and Neonatal Disease, Animal Reproduction Research Institute, Giza, Egypt.

**Keywords:** biofilm genes, mastitis, molecular identification, *Staphylococcus aureus*

## Abstract

**Background and Aim:**

Mastitis is considered a significant disease of lactating animals. There are new attitudes for recognizing genes responsible for causing this disease to overcome and change the manipulation of this problem. This study aimed to isolate and identify *Staphylococcus* *aureus* strains from mastitic bovine animals and detect some specific biofilm-forming genes (*icaA*, *icaD*, and biofilm-associated protein [*bap*] genes *clfA, fnbA, agrI, agrII, agrIII, agrIV*, and *cna*).

**Materials and Methods:**

A total of 121 mastitic milk samples were analyzed using biochemical tests (catalase test, oxidative-fermentative test, and coagulase test) and Gram stain. Multiplex polymerase chain reaction was applied to characterize biofilm genes (*icaA, icaD, bap, clfA*, and *fnbA*) in addition to (*agrI*, *agrII*, *agrIII*, *agrIV*, and *cna*).

**Results:**

Among the 121 milk samples, 35 staphylococci isolates were derived with an incidence of 28.92% (35/121); among them, 19 are coagulase positive. Ninety percent of the isolates had *ica* genes (*icaA and icaD*) while *bap* gene was not recognized in any isolate. In addition, the incidence of *fnbA*, *can, and* *clfA* was 89.5% each. The prevalence of *agr* specific groups (*agrI*, *agrII*, *agrIII*, and *agrIV*) was 78.9%, 52.6%, 10.5%, and 15.8%, respectively.

**Conclusion:**

This study concluded that *S. aureus* has variant mechanisms of pathogenicity to form biofilm devoid of carrying a specific gene.

## Introduction

Mastitis in bovine is the most widespread disease among dairy cattle. Consequently, it influences its production due to a combination of different factors such as host, climate, and infectious agents and reduces the quantity and quality of produced milk [1]. The primary cause of bovine mastitis is *Staphylococcus aureus*, responsible for a dramatic decrease in both quality and quantity of milk production, resulting in significant economic consequences in the dairy farms [2].

*S. aureus* strains have immense pathogenicity (e.g., encoding virulence factor genes and antibiotic resistance) and several ways to infect humans, such as through foodstuffs, skin infection during the milking process, and direct contact with contaminated fomites. Subsequently, animals producing food, especially cows, are a major route for *S. aureus* to enter the food chain [3]. Thus, identifying the virulence factors and mechanisms of this pathogen involved in such criteria is important. Furthermore, how they assist in adhering and colonizing the mammary glands’ epithelium cells, leading to persistence, successful foundation, and continuance in the host tissue. The most common virulence factors that aid staphylococci in adhering and colonizing the epithelium of the mammary gland are related to their capacity to form biofilms, resulting in loss of immunological defenses and frequent infections. Furthermore, staphylococcal adhesions have been shown to be necessary for binding host cells [4]. Biofilm formation may lead to continual contamination or infection because biofilm cells are highly resistant to hygiene measures, the effect of antimicrobial agents, and host immunity [5]. The process of biofilm formation by *Staphylococcus* spp. requires the contribution of different genes and proteins [6]. First, adherence of bacterial cells to a surface is initiated by a capsular antigen polysaccharide/adhesin (PS/A). Following that, growth occurs to create a multi-layered biofilm that induces *polysaccharide intercellular adhesin* (PIA) production. The intercellular adhesion operon is responsible for PIA and PS/A synthesis in staphylococcal species (*ica*), formed by the *icaA*, *icaB*, *icaC*, and *icaD* encoding genes as well as regulatory gene, *icaR*, carrying *icaA*, *icaB*, *icaC*, and *icaD* proteins [7].

Furthermore, *S. aureus* has many adhesins that play an important role in the onset of pathogenicity through the binding of host tissues that are considered essential factors of virulence. These adhesins include fibronectin-binding proteins (*fnbA* and *fnbB*), clumping factors (*clfA* and *clfB*), collagen-binding protein (*cna*), biofilm-associated protein (*bap*) [8], and collagen-binding protein (*cna*) [9], which are considered essential virulence factors in binding host cells, colonization, and invasion [10]. The accessory gene regulatory (*agr*) system is fundamental in *S. aureus* virulence gene expression. The *agr* operon, which includes the genes *agrA*, *agrB, agrC*, and *agrD*, controls more than 70 genes in *S. aureus*, 23 of which regulate infectivity [11]. Furthermore, *S. aureus* genes could be divided into four groups of (*agr I*, *agr II, agr III*, and *agr IV*) genes. They are different in their characteristics and occurrence in various geographical areas. Thus, it is necessary to determine the main types in each region [12].

This study aimed to isolate and identify *S. aureus* strains obtained from mastitic bovine animals and specify the involved genes in biofilm formation (*icaA, icaD, bap, clfA, fnbA, AgrI, AgrII, AgrIII, AgrIV*, and *cna*).

## Materials and Methods

### Ethical approval

This study was approved (no. 12020232/2019) by Ethical Committee for Medical Research at the National Research Centre, Egypt.

### Study period and location

The study was conducted from January to March 2020. The study was conducted at National Research Center, Dokki, Egypt. The samples were processed at the National Research center, Veterinary Research Division, Microbiology and Immunology Department Laboratory.

### Collection of samples

One hundred and twenty one affected quarter milk samples (quarter selected based on physical examination; appearance of inflammation as redness and swelling) were obtained from 40 cows suffered from mastitis and did not receive any medical treatment for 7-10 days in private farms in Giza Governorate, Egypt. Those farms did not implement the required hygienic measures to control mastitis and other infectious diseases. The milking process was performed using a traditional method.

Before the milk collection, the animals did not receive antibiotic treatment for at least 1 month. The collection of milk samples was conducted under complete aseptic conditions according to Oliver *et al*. [13].

### Isolation and identification of *S. aureus*

Every milk sample was cultured on two plates: Columbia Agar base with 5% defibrinated sheep blood (Oxoid, UK) and mannitol salt agar (Oxoid). The tested plates were incubated at 37±1°C for 24-48 h. According to colony morphology, Gram staining, in addition to catalase reaction, and oxidative-fermentative test, all isolates were identified as staphylococci. Coagulase test was used to characterize all *S. aureus* strains [14].

### DNA extraction

DNA milk samples were extracted using the QIAamp DNA Mini kit (Qiagen, Germany, GmbH) with some modifications (temperature adjustment at 25°C and pH set at 4.0) to the manufacturer’s instructions.

### Oligonucleotide primers

The utilized primers of polymerase chain reaction (PCR) from Metabion (Germany) are listed in Table-1 [8,15-19]. PCR was conducted in an Applied Biosystems 2720 thermal cycler (Applied Biosystems, USA).

**Table-1:** Primers sequences, target genes, amplicon sizes, and cycling conditions.

Target gene	Primers sequences	Amplified segment (bp)	Primary denaturation	Amplification (35 cycles)			Final extension	Reference

				Secondary denaturation	Annealing	Extension		
*16S rRNA*	CCTATAAGACTGGGATAAC TTCGGG	791	94˚C	94°C	55°C	72°C	72°C	[15]
	CTTTGAGTTTCAACCTTGCG GTCG		5 min	30 s	40 s	45 s	10 min	
*icaA*	CCT AAC TAA CGA AAG GTA G	1315	94°C	94°C	49°C	72°C	72°C	[16]
	AAG ATA TAG CGATAA GTG C		5 min	30 s	1 min	1 min	10 min	
*icaD*	AAA CGTAAG AGA GGT GG	381	94°C	94°C	49°C	72°C	72°C	
	GGC AAT ATG ATC AAGATA		5 min	30 s	40 s	40 s	10 min	
*Bap*	CCC TAT ATC GAA GGT GTA GAA TTG	971	94°C	94°C	62°C	72°C	72°C	[8]
	GCT GTT GAA GTT AAT ACT GTA CCT GC		5 min	30 s	40 s	50 s	10 min	
*ClfA*	GCAAAATCCAGCACAACAG GAAACGA	638	94°C	94°C	55°C	72°C	72°C	[15]
	CTTGATCTCCAGCCATAAT TGGTGG		5 min.	30 sec.	40 sec.	45 sec.	10 min.	
*FnbA*	CATAAATTGGGAGCAGCAT CA	127	94°C	94°C	58°C	72°C	72°C	[17]
	ATCAGCAGCTGAATTCCCA TT		5 min	30 s	30 s	30 s	7 min.	
	GTAAATGCACTTGCTTCAG GAC							
*AgrI*	Pan: ATG CAC ATG GTG CAC ATG C	441	94°C	94°C	55°C	72°C	72°C	[18]
	GTC ACA AGT ACT ATA AGC TGC GAT		5 min	30 s	40 s	45 s	10 min	
*AgrII*	Pan: ATG CAC ATG GTG CAC ATG C	575	94°C	94°C	55°C	72°C	72°C	
	TAT TAC TAA TTG AAA AGT GGC CATAGC		5 min	30 s	40 s	45 s	10 min	
*AgrIII*	Pan: ATG CAC ATG GTG CAC ATG C	323	94°C	94°C	55°C	72°C	72°C	
	GTA ATG TAA TAG CTT GTA TAA TAA TAC CCA G		5 min	30 s	40 s	40 s	10 min	
*AgrIV*	Pan: ATG CAC ATG GTG CAC ATG C	659	94°C	94°C	55°C	72°C	72°C	
	CGA TAA TGC CGT AAT ACC CG		5 min	30 s	40 s	45 s	10 min	
*Can*	GTCAAGCAGTTATTAACACCAGA C	423	94°C	94°C	55°C	72°C	72°C	[19]
	AATCAGTAATTGCACTTTGTCCAC TG		5 min	30 s	40 s	45 s	10 min	

### PCR products

PCR products were separated by electrophoresis at room temperature (25°C) on 1.5% agarose gel (AppliChem, Germany, GmbH) in 1× Tris-borate-EDTA buffer with gradients of 5 V/cm. A gel documentation system (Alpha Innotech, Biometra, Germany) was used to photograph the gel (Gel documentation system; Biometra, Germany) and the data were evaluated using a computer software (Genesys image capture, Biometra, Germany).

## Results and Discussion

Bovine mastitis is a significant disease in lactating herds worldwide [20]. *S. aureus* is considered the most common causative agent, leading to more virulent mastitis in cows. It possesses the greatest risk in dairy production in many countries [21].

**Figure-1:**
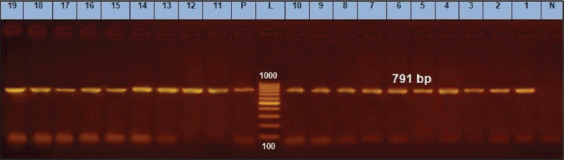
*16S rRNA* gene at 791bp; Lanes (1-11) are positive, Lane L: 1000 bp ladder, N: (negative control) and P: (positive control).

In the current study, a total of 35 staphylococci isolates have been isolated with a prevalence of 28.92% (35/121). The identified prevalence of *S. aureus* and Coag–ve staphylococci other than *S. aureus* was 57.14% (20/35) and and 42.85% (15/35). All staphylococci isolates were confirmed using *16S rRNA* gene, as shown in Figure-1.

*S. aureus* isolated from subclinical mastitis cow was 36% [22] and less than the prevalence (6.5%) detected by Haltia *et al*. [23]. According to this study, the high prevalence of *S. aureus* may result from contaminated milk utensils and Milker’s hands.

The possibility of *S. aureus* infection occurring is related to its capacity to release different factors of virulence that contribute to the invasion of the bacteria [24]. The formation of biofilm increased the virulence of *S. aureus*. In addition, strains can create biofilms that possess higher antibiotic tolerance, antiseptics, and poor environmental conditions [25]. The genes of *ica* are responsible for slime formation in *S. aureus* by controlling PIA production. It can also determine the ability of *S. aureus* strains to generate biofilm.

**Figure-2:**
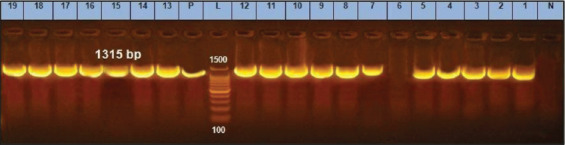
*icaA* gene at 1315bp; Lanes 6 is negative, Lane L: 1000 bp ladder, N: (Negative control) and P: (Positive control).

**Figure-3:**
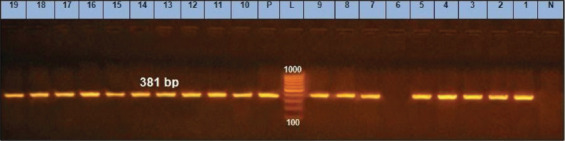
*icaD* gene at 381 bp; Lanes 6 is negative, Lane L: 1000 bp ladder, N: (Negative control) and P: (Positive control).

Namvar *et al*. [26] found that *S. aureus* could not generate biofilm unless strains were positive for the *ica D* gene. Strains posing the *ica* ADBC group were likely to generate biofilm [27]. Among 90% of *S.aureus* isolates (Table-2), *ica* genes (*ica A* and *ica D*) were detected (Figures-2 and 3).

**Table-2:** Polymerase chain reaction results for biofilm genes.

Coagulase positive staphylococcus sample	*16S rRNA*	*icaA*	*icaD*	*fnbA*	*can*	*bap*	*clfA*	*agrI*	*agrII*	*agrIII*	*agrIV*
1	+	+	+	+	+	–	+	–	+	–	+
2	+	+	+	+	+	–	+	+	–	–	–
3	+	+	+	+	+	–	+	+	–	–	+
4	+	+	+	–	–	–	–	–	–	–	–
5	+	+	+	+	+	–	+	+	–	+	–
6	+	–	–	–	–	–	–	–	–	–	–
7	+	+	+	+	+	–	+	–	+	–	+
8	+	+	+	+	+	–	+	+	+	–	–
9	+	+	+	+	+	–	+	+	+	–	–
10	+	+	+	+	+	–	+	+	–	–	–
11	+	+	+	+	+	–	+	+	+	–	–
12	+	+	+	+	+	–	+	+	–	–	–
13	+	+	+	+	+	–	+	+	+	–	–
14	+	+	+	+	+	–	+	+	+	–	–
15	+	+	+	+	+	–	+	+	–	+	–
16	+	+	+	+	+	–	+	+	+	–	–
17	+	+	+	+	+	–	+	+	–	–	–
18	+	+	+	+	+	–	+	+	+	–	–
19	+	+	+	+	+	–	+	+	+	–	–

These results almost agree with other findings as *ica* genes were identified in all isolates [3]. While Gowrishankar *et al*. [28] detected those isolates of *S*. *aureus* in India carry *ica* genes in a percentage of 84.13%. In Mexico, Avila-Novoa *et al*. [29] identified the genes in 52.3% of isolates.

**Figure-4:**
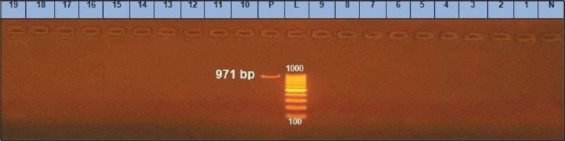
*bap* gene at 971bp; Lane (1-19) are negative, Lane L: 1000 bp ladder, N: (Negative control) and P: (Positive control).

The *bap* gene implicates biofilm formation by promoting primary attachment and adhesion to inert and live surfaces [30]. This study showed that all the tested strains (100%) were negative for the *bap* gene (Figure-4).

According to Vautor *et al*. [31], the absence of *bap* indicates that the ica-dependent pathway is predominantly responsible for adhesion and biofilm development in strains. Bissong *et al*. [32] found that the occurrence of the *bap* gene was limited (12, 15.6%). Li *et al*. [33] identified *bap* gene in 43.9% of *S. aureus* strains biofilm producers, proving the significance of *bap* gene in biofilm production. Our results are in agreement with Xu *et al*. [34], who were unable to detect *bap* gene in *S. aureus* recovered from subclinical mastitic cow. Khoramrooz *et al*. [35] and Darwish and Asfour [36] detected expression of *bap* gene in 5% and 2.5% among obtained isolates, respectively. Our results showed that the *ica*- gene process could sometimes be essential for attachment and formation of biofilm among isolated strains; this can be a possible explanation for our finding.

**Figure-5:**
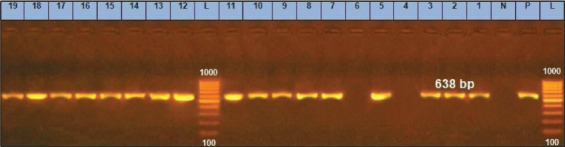
*clfA* gene at 638bp; Lanes (4 and 6) are negative, Lane L: 1000 bp ladder, N: (Negative control) and P: (Positive control).

**Figure-6:**
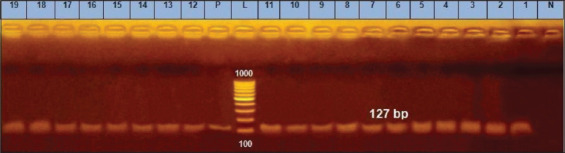
*fnbA* gene at 127bp; Lane L: 1000 bp ladder, N: (Negative control) and P: (Positive control).

**Figure-7:**
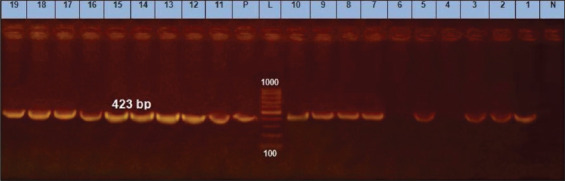
*cna* gene at 423 bp; Lanes (4 and 6) are negative, Lane L: 1000 bp ladder, N: (Negative control) and P: (Positive control).

The *fnbA* genes appear to be necessary for bacterial invasion and adhesion, and they may be associated with their ability to form biofilms. The incidence of expression of the surface protein genes for *S. aureus* (*fnbA*, *can*, and *clfA*) was 89.5% for each gene as reported in Table-2; these results demonstrated resemblance and minor variations to earlier studies; Peerayeh *et al*. [37] revealed that *clfA* and *fnbA* (Figures-5 and 6) encoding genes had been found in each of the tested isolates (20 strains), with *can* gene are being found in 20% of identified isolates(Figure-7). In contrast, Ote *et al*. [38], and Ikawaty *et al*. [39] observed a higher prevalence of *can* gene (31.9% and 49%, respectively).

**Figure-8:**
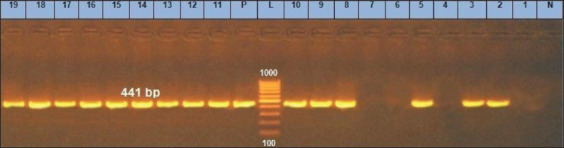
*agrI* gene at 441bp; Lanes (1, 4, 6, 7) are negative, Lane L: 1000 bp ladder, N: (Negative control) and P: (Positive control).

**Figure-9:**
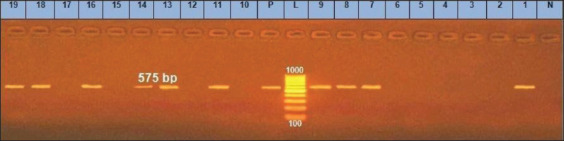
*agrII* gene at 575bp; Lanes (2, 3, 4, 5, 6, 10, 12, 15, 17) are negative, Lane L: 1000 bp ladder, N: (Negative control) and P: (Positive control).

**Figure-10:**
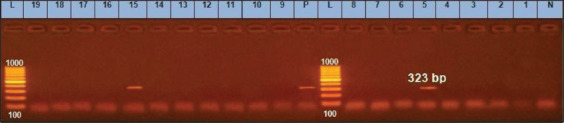
*agrIII* gene at 323bp; Lanes (5 and 15) are positive, Lane L: 1000 bp ladder, N: (Negative control) and P: (Positive control).

**Figure-11:**
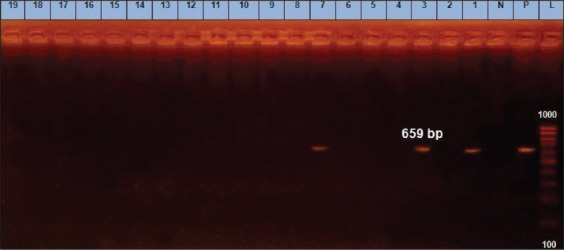
*agrIV* gene at 659bp; Lanes (1, 3, 7) are positive, Lane L: 1000 bp ladder, N: (Negative control) and P: (Positive control).

*S. aureu*s strains have been divided into four categories, *agrI* to *agrIV*, [18] based on differences in their *agr* genes. The *agr*-encoded quorum-sensing system’s important function in virulence regulation makes it an appropriate target for antimicrobial drug development. According to Table-2, the prevalence of *agr* specificity groups (*agrI, agrII, agrIII*, and *agrIV*) were 78.9%, 52.6, 10.5%, and 15.8%, respectively. According to Figures-8 to 11, our results showed that *agr*I was the most common type found in isolated *S. aureus*. Javdan *et al*. [40], and Cheraghi *et al*. [41], stated that the most dominant type was *agr* Type I. It is said that definite groups of *agr* in *S. aureus* are implicated in certain diseases, such as isolates that possess *agrI* are related to bacteremia and persistent diseases [42]. The development of biofilm is a complex process involving numerous factors such as poor hygienic measures and poor management of milking practices.

## Conclusion

It can be concluded that the variant mechanisms of pathogenicity induced by *S. aureus* to form biofilm without needing a specific gene. It is necessary to identify the presence of genes related to biofilm production as formation of biofilm results in attachment to glandular udder tissue and biomaterials, thus increasing the virulence of bacteria. Besides, the biofilm’s existence increases the bacterial resistance to antibiotics, consequently complicating its treatment. This study presents preliminary results for additional in-depth prospect studies. Finally, good hygienic measures and habits, in addition to good management of milking practices, can reduce the incidence of *S. aureus* mastitis.

## Authors’ Contributions

RHE and EGS: Collected the samples, applied the practical work including isolation of *S. aureus* and identification of obtained isolates. ESI, WHE and AAA: Planned the work, applied the practical work including isolation of *S. aureus* and identification of obtained isolates, PCR and revised the manuscript. SMD: Carried out PCR and drafted the manuscript. NSA: Revised the manuscript. All authors read and approved the final manuscript.
